# Editorial: Understanding radiation events through health and policy: applying historical, cultural, and social perspectives to science and advocacy

**DOI:** 10.3389/fpubh.2024.1476393

**Published:** 2024-08-28

**Authors:** Tomoko Y. Steen, Armin D. Weinberg

**Affiliations:** ^1^Department of Microbiology and Immunology, Georgetown University School of Medicine, Washington, DC, United States; ^2^Department of Medicine, Baylor College of Medicine, Houston, TX, United States; ^3^Department of Kinesiology, Rice University, Houston, TX, United States

**Keywords:** biological effects of ionizing radiation, ABCC, radio particles, gut-brain axes, food steralization, Fukushima nuclear accident, nuclear power plant, radiation

## 1 Introduction

While one can debate just when the world began paying attention to radiation related issues—there is consensus that the nuclear age opened in 1945 with the Trinity Test in Nevada. The deployment of atomic bombs in Hiroshima and Nagasaki stunned the world with the level of destruction that nuclear power could produce and the health effects of the unleased radiation. The fear of ionizing radiation has continued globally. The editors of this Research Topic have long-term associations with the impacts of ionizing radiation on humans and other living organisms as an internationally known cancer specialist or clinical pharmacologist in Nagasaki, or as a population geneticist and microbiologist in Japan and the United States, in addition to being a historian of science and a policy expert.

## 2 History

Part 1 of this Research Topic starts with an opinion piece and biographical sketch, *William Jackson Schull and mutation studies on human cohorts* by Steen. The name William Jackson Schull, as he had been a leading human geneticist who worked on cases of atomic bomb survivors for many decades, is often associated with (almost synonymous to) studies of the biological effects of atomic radiation in Hiroshima and Nagasaki. His life's work had been on C*hildren of atomic bombs* and “lifespan studies,” i.e., long term studies on the effects of atomic radiation on survivors in those cities. Professor Schull's work is beyond epidemiology and genetics. He studied the history and sociology of people in those cities, including the cousin marriage in Japan and practices of the hidden Christian populations. He analyzed the atomic radiation effects from historical, cultural, and sociological perspectives and associated with these populations while accumulated scientific data. His multidisciplinary approaches to epidemiology of atomic survivors are the primary motivation for us to create this Research Topic. Professor Schull often said that the fear of ionizing radiation can be assessed correctly by having accurate data.

## 3 Nuclear power plant safety

While many hear about the numerous coal-fueled power plants being built in China few recognize China is one of the world's leaders in nuclear power production as well. Two articles report on ionizing radiation studies on the Sanmen Nuclear Power Plant built in Sanmen County in Taizhou, Zhejiang (China). As China heavily depends on nuclear power and building nuclear power plants, guaranteeing the plants' safety to their residents is the priority. Ren et al. presented “*Assessment of radiation exposure and public health before and after the operation of Sanmen nuclear power plant*.” Looking back the cases of the Chornobyl and Fukushima nuclear accidents, availability of the data before the accidents is often not possible, but the authors successfully gathered health records of residents even before the plant was operated in 2014 and compared to potential health effects of residents from the residual ionizing radiation from the power plant throughout to 2021. They found no significant health effects after the plant's operation.

Wang et al. “*Levels, sources, variations, and human health risk assessment of* ^90^*Sr and* ^137^*Cs in water and food around Sanmen Nuclear Power Plant (China) from 2011 to 2020*” monitored release of two critical radioisotopes (Strontium 90 and Cesium 137) from the same Sanmen power plant. Again, they found little significance between before (2011) the operation and after (2020) the nuclear power plant's operation. A noteworthy comment in the paper, however, is that they identified radioisotopes from nuclear testing from the 1950's to the 1970's during this study. These two scientific data would support the safety of the newer nuclear power plant, specifically the Sanmen power plant. According to their data, the plant has been safely operating without releasing significant amounts of nuclear isotopes and has had no significant effects on the health of residents. These studies can provide a baseline should issues arise in future years that will provide added value to any conclusions and reduce the fear toward nuclear power plants.

As future population's will undoubtably be exposed to the effects of radiation we must strive to build better systems to monitor the numerous elements beyond “isotopes!” The final article by Kitazawa et al., *Disaster-related deaths with alcohol-related diseases after the Fukushima Daiichi nuclear power plant accident: case series* has a case study on the survivors of the Fukushima nuclear accident. The Fukushima population was given conflicting information on the biological effects of ionizing radiation after the accident, and the fear of radiation still strong among the population. In addition, they had to evacuate multiple times due to uncertainty of the levels of ionizing radiation effects in the areas. Alcohol abuse is one of the outcomes of the fear of the biological effects of ionizing radiation in this case. The authors closely monitored individuals after they experienced ionizing radiation exposure in Fukushima and multiple evacuations. They try to assess if the fear itself and thus depression brought death of these individuals.

## 4 Food safety and radiation

At a time when we continue to seek safe alternatives to carbon-based energy sources to meet the needs of an ever-increasing global population, the concurrent need for food availability, stability and safety may lie in food irradiation technology. The study of Buczkowska et al., *The attitude of Polish consumers toward food irradiation as one of the methods of food preservation* is another unique approach to studying public understanding of radiation use. Food irradiation methods have been used since the 1950's, and internationally, the public has rather negative perception of such methods as again it is based on the fear toward ionizing radiation. However, this study concludes that in Poland populations shows less fear if they are more educated and younger. Between men and women, women have positive attitudes toward consumption of irradiated food. The authors claimed that educating the population about irradiated food through this study, the number of positive attitudes increased. This study shows that solid scientific information and data would avoid unnecessary fear toward ionizing radiation.

## 5 Conclusion: radiation policy and scientific data

Considering the gut and brain axis, an individual's immune system is directly related to mental state and vise versa. For this reason, it is also crucial to continue to produce accurate data on the health effects of ionizing radiation including the connection to mental state and fear. Further studies are necessary in this area. International standards for the safety of ionizing radiation and health effects have been produced by several organizations, including the International Atomic Energy Agency (IAEA), International Commission on Radiation Protection (ICRP) and US regulator such as, Nuclear Regulatory Commission (NRC), while the numbers are not necessary the same. General public has a right to obtain the accurate number for their safety level and the number should be updated regularly as new data with a variety of studies are produced. Establishing an international policy for radiation safety with the globally agreed data sets is key to avoid unnecessary fear. Part 2 of this series further discuss these Research Topics.

